# Speeding Up the Write Operation for Multi-Level Cell Phase Change Memory with Programmable Ramp-Down Current Pulses

**DOI:** 10.3390/mi10070461

**Published:** 2019-07-08

**Authors:** Chenchen Xie, Xi Li, Houpeng Chen, Yang Li, Yuanguang Liu, Qian Wang, Kun Ren, Zhitang Song

**Affiliations:** 1 Schools of Microelectronics, University of Chinese Academy of Sciences, Beijing 100049, China; 2 State Key Laboratory of Functional Materials for Informatics; Shanghai Institute of Microsystem and Information Technology, Chinese Academy of Sciences, Shanghai 200050, China

**Keywords:** multi-level cell, phase change memory, programmable ramp-down current pulses

## Abstract

Multi-level cell (MLC) phase change memory (PCM) can not only effectively multiply the memory capacity while maintaining the cell area, but also has infinite potential in the application of the artificial neural network. The write and verify scheme is usually adopted to reduce the impact of device-to-device variability at the expense of a greater operation time and more power consumption. This paper proposes a novel write operation for multi-level cell phase change memory: Programmable ramp-down current pulses are utilized to program the RESET initialized memory cells to the expected resistance levels. In addition, a fully differential read circuit with an optional reference current source is employed to complete the readout operation. Eventually, a 2-bit/cell phase change memory chip is presented with a more efficient write operation of a single current pulse and a read access time of 65 ns. Some experiments are implemented to demonstrate the resistance distribution and the drift.

## 1. Introduction

Data is the most competitive resource in the twenty-first century and its heat has never been cut down. Especially with the advent of the big-data era and artificial intelligence, a massive amount of data needs to be processed and saved, which undoubtedly brings unprecedented challenges to the memory market. Phase change memory (PCM), one of the most promising novel non-volatile memories, attracts much attention due to its prominent performances. Compared with the mainstream flash memory, PCM has an excellent reliability below 20 nm technology [1] and its scaling is more favorable when the NMOS (N-Metal-Oxide-Semiconductor) devices are replaced by the FinFETs [2]. What is more, the large resistance contrast between amorphous and crystalline states (typically three or four orders of magnitude) in the memory cell means that PCM has more potential in multi-level cell (MLC) storage, which is a crucial feature for reducing the cost-per-bit and increasing the memory capacity. The MLC PCM can also be used in artificial neural networks as synapses, which provides a promising solution for energy-efficient artificial neural networks (ANNs) [3,4]. Therefore, the research on multi-level phase change memory cell storage is of great significance to the future development of the non-volatile memory market.

However, the realization of MLC PCM still faces several challenges. First of all, new program and read schemes should be specifically proposed since the intermediate states that represent the extra bits are avoided as much as possible in conventional phase change memory. Then, the corresponding circuits need to be well-designed, taking both performance and efficiency into consideration. Finally, as a novel storage technology that improves the capacity at the expense of reliability, a physical issue called “resistance drift” may produce severe reliability problems as it reduces the separation between adjacent levels.

Previous research has made some progress in multi-level cell phase change memory technology. T. Nirschl et al. came up with a novel multi-level program algorithm based on write and verify cycles to produce highly optimized resistance distributions in PCM [5]. G. F. Close et al. analyzed the impact of noise in multi-bit PCM from different levels [6]. N. Papandreou et al. introduced advanced iterative programing schemes for multilevel storage in PCM to achieve a high robustness to cell variability and low latency [7]. A new cell-state metric was proposed by N. Papandreou et al. to obtain larger level contrast in PCM and reduce the sensitivity to drift [8]. A 256-Mcell PCM chip operating at 2+ bit/cell, which means that the actual capacity can reach 512 Mb, was presented by Gael F. Close et al. [2]. Milos Stanisavljevic et al. discussed the storage and retention of data in MLC PCM at elevated temperatures [9].

This paper starts with the principle of multi-level cell storage in phase change memory and explores the relationship between the resistance distribution of a memory cell and the program current pulses. Then, a PCM memory chip that demonstrates an MLC operation at 2-bit/cell is presented. The entire work involves the program scheme of multi-level storage, chip structure, circuit realization, and the results of the simulation and experiments. Eventually, a 4-Mcell PCM is expanded to an 8 Mb capacity by multi-level storage technology.

The remainder of this paper is organized as follows: Section 2 briefly introduces the basic characteristics of PCM and discusses the fundamental principles of MLC PCM; chip architecture, specific write-read schemes combined with the circuit implement are demonstrated respectively in Section 3; Section 4 presents the results of experiments and the simulation; and conclusions are drawn in Section 5.

## 2. Phase Change Storage Technology 

### 2.1. Basic Characteristics of Phase Change Memory

The basic principle of phase change storage is the chalcogenide phase change materials’ (typical Ge_2_Sb_2_Te_5_, GST) reversible transformation between two different phases (amorphous and crystalline phase) by internal structure changes [10,11]. The great difference in electrical properties between two phases makes it possible to store binary data: the amorphous phase with a high resistance usually represents ‘0′ and the crystalline one represents ‘1′, with a lower resistance. 

**Figure 1:**
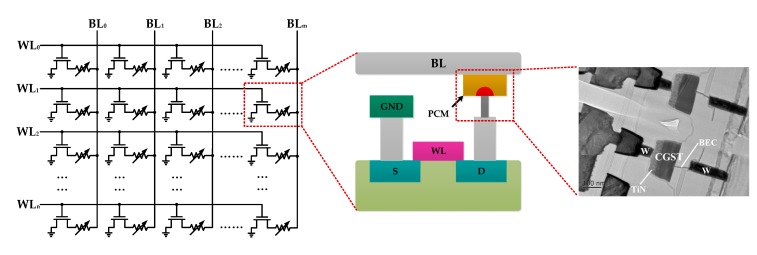
The storage array of phase change memory and the TEM image of a phase change memory (PCM) cell.

Figure 1 shows the storage array of phase change memory and the transmission electron microscope (TEM) image of a PCM cell. Each cell consists of a layer of phase change material sandwiched between a top and bottom electrode and an access device, which is typically a MOSFET (Metal-Oxide-Semiconductor Field-Effect Transistor). Phase transformation is usually performed by applying programming pulses (voltage or current) to the bit line of the selected phase change memory cell. The Joule heat generated by the current flowing through the phase change memory cell causes the phase change material to melt and quench, thus producing mushroom-shaped amorphous phase in the crystalline phase, as shown in Figure 1.

**Figure 2:**
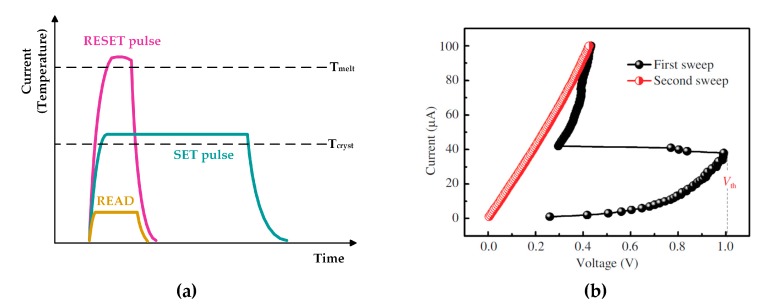
(**a**) Program and read operations of phase change memory (PCM); (**b**) the threshold switching phenomenon of chalcogenide compounds.

Figure 2a shows the program and read pulses of PCM. The RESET program operation from crystalline to amorphous phase is usually performed by a rectangular current pulse with a large amplitude and narrow width. In order to make the phase change material quench to amorphous phase, the RESET pulse must have an abrupt trailing edge. As for SET operation, a wider current pulse with a lower amplitude is usually used to heat the cell to its crystallization temperature until it becomes crystalline phase. The typical current –voltage (I–V) characteristics of a PCM cell are shown in Figure 2b. With the increase of the voltage applied to the memory cell, the current flowing through the amorphous phase cell increases slowly. Until the voltage reaches a certain value V_th_, however, the resistance of the phase change memory cell drops sharply, which is known as the threshold switching phenomenon of chalcogenide compounds. Therefore, during the reading process, the voltage applied to the addressed cell must be kept well below V_th_ to ensure the accuracy of the read-out data.

### 2.2. Multilevel-Cell Storage

**Figure 3:**
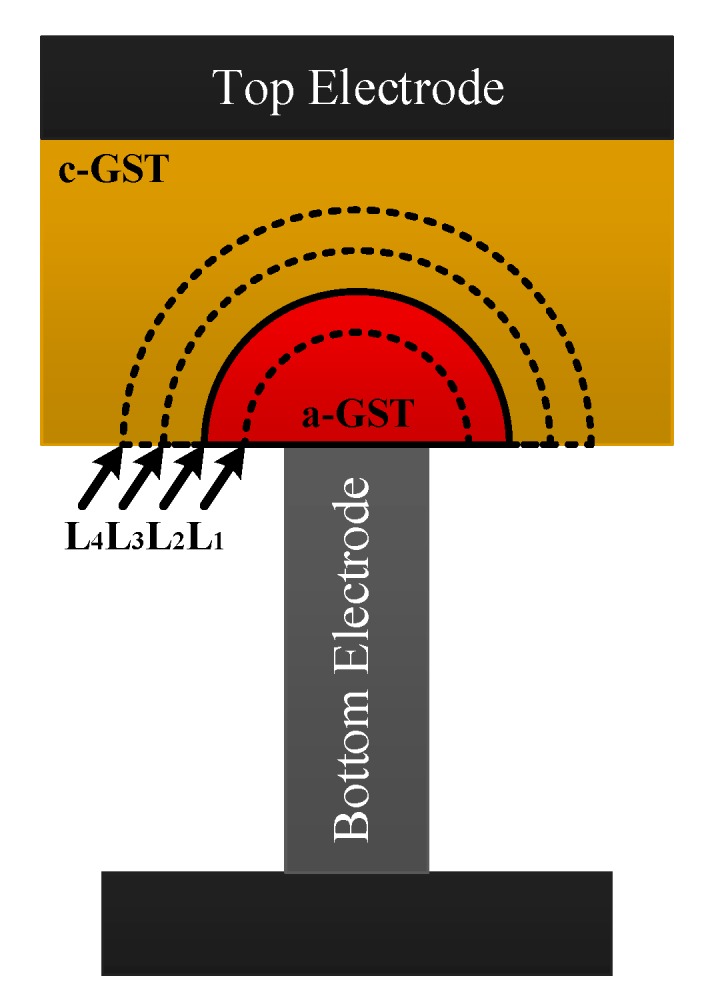
The sectional view of the phase change material layer with different amorphous regions.

In PCM, the essential difference between two opposite phases is that the amorphous degree of the phase change material layer is different; in other words, the amorphous region and its thickness are different. The electrical resistance of the cell is only utilized to measure these differences. In conventional applications, intermediate states are usually avoided in the PCM cell to guarantee the accuracy of data storage. However, by changing some parameters, like the amplitude, of programming pulses, the PCM cell can be stabilized in the intermediate state, which is the basic state for multilevel storage in PCM [8]. What is more, the large resistance contrast, which is around three to four orders, between amorphous and crystalline phase leaves a sufficient margin for the realization of intermediate states. Figure 3 shows the sectional view of the phase change material layer with different amorphous regions.

**Figure 4:**
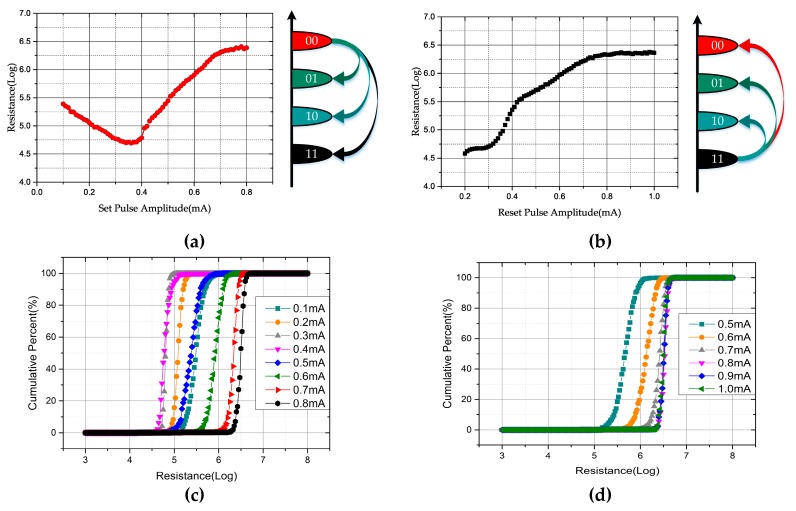
The characteristic programming curve of the phase change memory (PCM) cell resistance as a function of pulse amplitude for (**a**) RESET initialization and (**b**) SET initialization; resistance distribution for (**c**) RESET initialization and (**d**) SET initialization.

When studying the programming conditions for realizing intermediate states, the initial state of the PCM cell should be considered. Figure 4a,b show the characteristic programming curve of the PCM cell resistance as a function of pulse amplitude. For the case where the initial state is high resistance and the programming operations are performed with SET pulses of different amplitudes, with the increase of the pulse amplitude, the resistance of the PCM cell first decreases and then increases. Taking 0.35 mA as the demarcation point, the curves before and after it both show some linearity. As for the other case, the overall curve does not show linearity, but it increases monotonously with the increase of the SET pulse amplitude. However, if the curve is piecewise analyzed, the part whose amplitude is between 0.42 mA and 0.7 mA also has a certain linearity. As shown in Figure 4c,d, whether in terms of the resistance distribution range or its consistency, using the SET operation to program memory cells is a better scheme for PCM multilevel storage.

## 3. Multilevel Cell Phase Change Memory Chip

### 3.1. Chip Architecture

**Figure 5:**
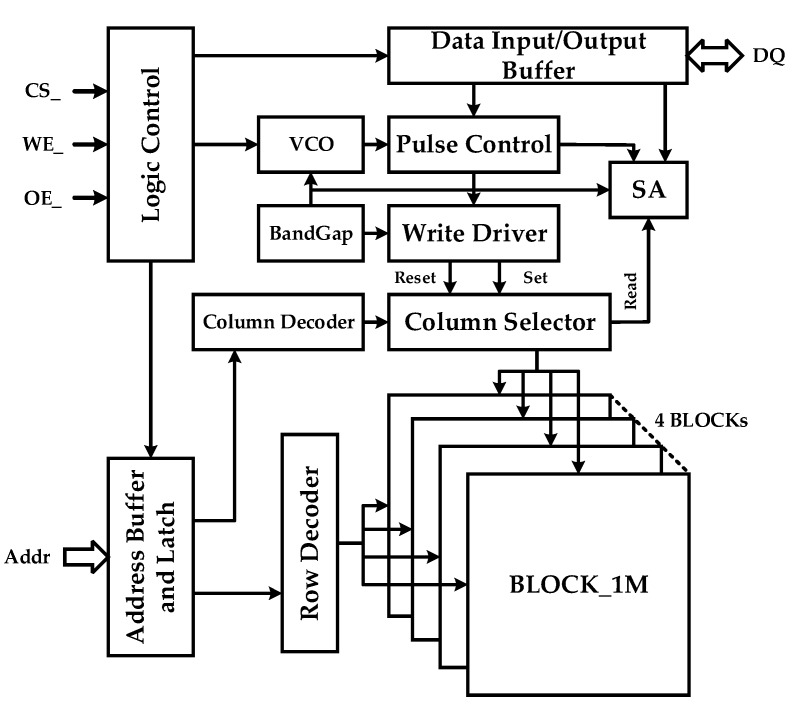
The architecture framework of the 4 M 2-bit/cell phase change memory chip.

**Figure 6:**
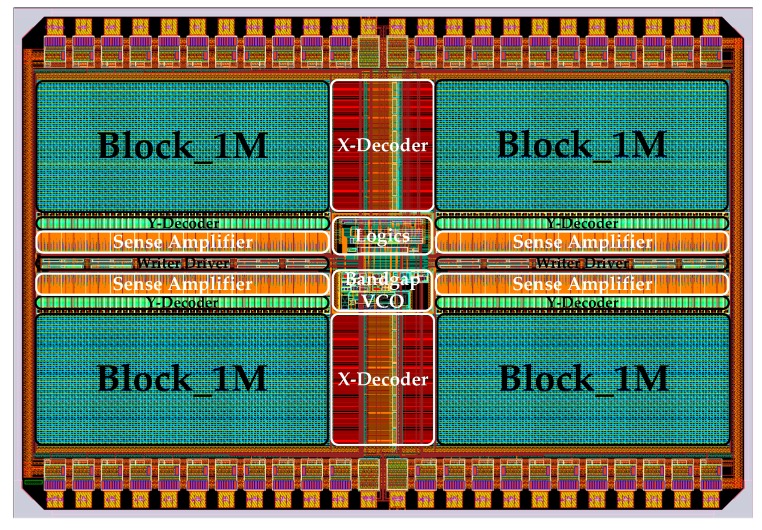
The layout of the 4 M 2-bit/cell phase change memory chip.

The overall framework of the 4 M 2-bit/cell phase change memory chip, which is shown in Figure 5, includes the following modules: PCM Storage Array, Row Decoder, Column Decoder, Column Selector, BandGap, Writer Driver, Voltage Controlled Oscillator (VCO), Pulse Control, Sense Amplifier, Logic Control, Address Buffer and Latch, Data Input/Output Buffer et al. The entire PCM Storage Array is divided into four 1 M cell blocks. The Row and Column Decoders locate the addressed memory cells according to the address signal saved in the Address Latch. BandGap and VCO generate the corresponding reference and clock signal on the basis of configuration parameters. Then, the Logic Control Module converts the external control signals, such as CS_, WE_, and OE_, into the internal read-write command to control the Write Driver and Sense Amplifier. Finally, the written and readout data interact with peripheral devices through the Data I/O Interface. Figure 6 shows the layout of the chip. Compared with traditional phase change memory, the biggest difference of MLC PCM lies in the read-write scheme and the specific circuit implementation, which will be covered in the flowing two subsections. 

### 3.2. Program Scheme and Circuit

From the analysis in the previous section, it can be seen that the broader resistance distribution can be obtained if the high-resistance PCM cells are operated with rectangular current pulses of different amplitudes. However, due to the process mismatch and energy loss in the bit line, the memory cells in the array may not achieve the same resistance level under the same pulse operation. To minimize the impact caused by cell variety, Samsung and STMicroelectronics propose “ASQ technology” [11] and “SET-Sweep Programming” [12], respectively, both of which are designed to extend the crystalline time of the PCM cells. Based on the same principle, a programmable ramp-down current pulse scheme is adopted to achieve a better cell resistance distribution.

**Figure 7:**
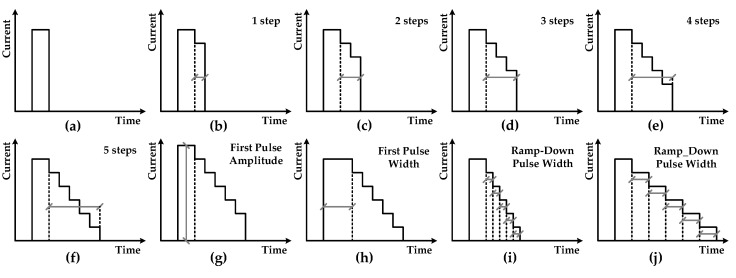
Programmable ramp-down current pulse for SET operation: (**a**) is a rectangular SET pulse without a ramp-down edge; (**b**–**f**) are ramp-down pulses with 1/2/3/4/5 steps, respectively; (**g**) is a five-step ramp-down pulse with a larger initial amplitude; (**h**) is a five-step ramp-down pulse with a larger initial width; (**i**,**j**) are five-step ramp-down pulses with different widths.

As shown in Figure 7, the descending edge of the slope current is achieved by constructing a finite number of ramp current pulses. Furthermore, in order to further obtain the optimal operating parameters of PCM cells related to the process, the initial height, initial width, and number and width of ramp current pulses are all adjustable. 

**Figure 8:**
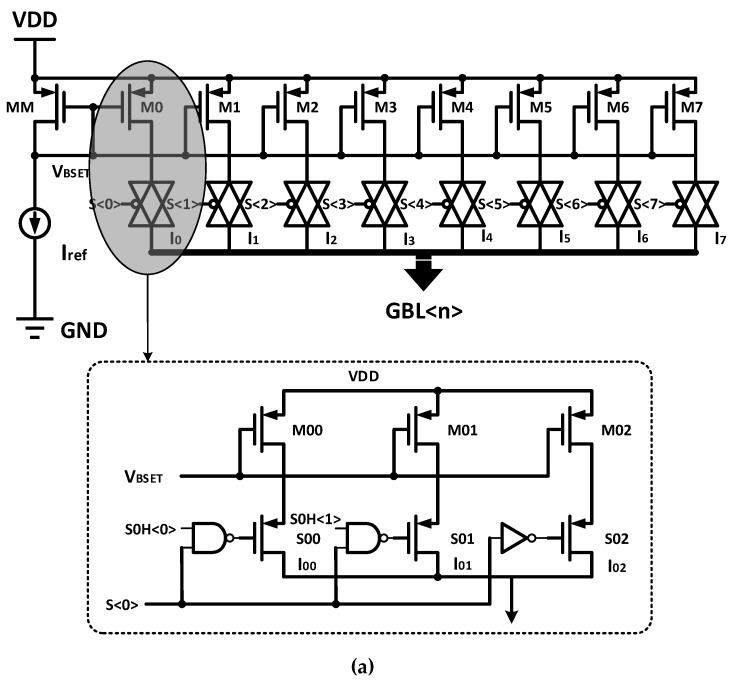

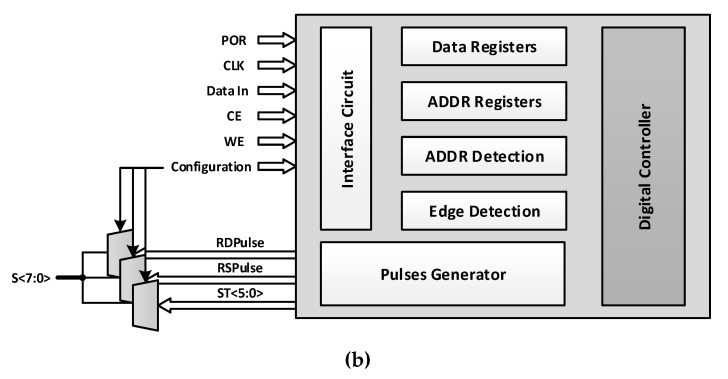
(**a**) Ramp-down current pulse generator circuit; (**b**) control circuit block diagram of the pulse generator.

To achieve the above scheme, the ramp-down current pulse generator circuit designed in this paper is shown in Figure 8a. The generator consists of eight current mirrors. During the SET programming process, the control switches S<0>~S<7> are turned on or off sequentially according to a certain order, and the SET current pulse with a specific shape can then be generated. The slope of the descent edge can be changed by controlling the opening time of each current source. In addition, in order to facilitate adjustment, a number of switches are designed in each current source, as shown in the lower half of Figure 8a. Four different amplitudes can be obtained by adjusting the combination of signal S0H <1:0>, and the height of each pulse in the ramp-down current can then be adjusted. Considering the high voltage on the bit line during the write operation, the transmission gate is implemented by a single PMOS, which can reduce the wiring of the layout and save the area at the same time. Figure 8b shows the control circuit block diagram of the pulse generator. The external signals are transformed into three kinds of control signals: RDPulse, RSPulse, and ST<5:0>, corresponding to READ, RESET, and SET operations, respectively. 

### 3.3. Readout Scheme and Circuit

The readout scheme of phase change memory is essentially adopted to utilize a specific circuit to measure the resistance of the memory cell. When the cell resistance is greater or less than the specific resistance value R_H_ or R_L_, the readout circuit outputs different digital levels respectively. The resistance interval R_L_~R_H_ is called the readout window of PCM. Generally, we choose R_REF_ = (R_L_ + R_H_)/2 as the reference resistance of the readout circuit. For MLC PCM with multi-bit stored in each cell, more readout windows need to be set up. In this paper, a readout scheme for 2-bit/cell phase change memory with an optional reference source is proposed, and the whole readout process is divided into two read operations: high-bit and low-bit readouts.

According to Ohm’s law, the resistance value of the PCM cell can be distinguished by two kinds of readout circuits: a current-bias voltage readout circuit and voltage-bias current readout circuit. By applying a constant current to the memory cell, the current-bias voltage readout circuit generates a reading voltage according to the cell resistance value. The voltage comparator then compares the reading voltage with the reference voltage to complete the cell resistance discrimination and output the logic level “0” or “1”. Correspondingly, the current-bias voltage readout circuit applies a certain voltage to the memory cell, and then compares the generated current with the reference current and outputs the logic level. However, due to the threshold effect of PCM and parasitic capacitance of the storage array, the realization of the current-bias voltage readout circuit is not realistic in practical applications.

**Figure 9:**
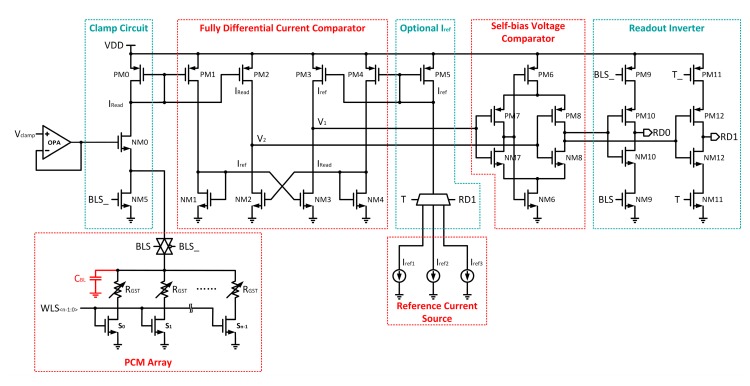
Fully differential high-speed readout circuit with an optional reference current source.

Figure 9 shows the fully differential high-speed readout circuit, which is based on the voltage-bias current readout scheme, included in this paper. The whole readout circuit can be divided into five parts: Clamp Circuit, Fully Differential Current Comparator, Optional I_ref_, Self-bias Voltage Comparator, and Readout Inverter. The Clamp Circuit controls the bit line voltage to V_clamp_–V_th0_ with a single transistor NM0. By setting V_clamp_ and V_th0_ reasonably, the bit line voltage can be limited under the threshold voltage of the PCM cell. This approach has a great bandwidth and can provide a fast clamping operation. Furthermore, in order to avoid the effect of path parasitic charge on the PCM cells during the whole read operation, a discharge transistor NM5 is added to the readout circuit. The fully differential current comparator is composed of two sets of current mirrors which are cross-coupled. It can quickly respond to the difference between I_read_ and I_ref_ and amplify them into differential voltage signals V_1_ and V_2_. Since there are multiple readout windows when reading each cell, the reference current source is designed to be optional. Firstly, three standard reference currents that can be changed by adjusting configuration parameters are generated by the bias circuit module inside the chip. Then, the high-bit reference current source is selected for the first read operation, and the reference current is mirrored into the current comparator through the current mirror composed of PM5. Finally, the selection of the current reference source during the second read operation is determined by the logic circuit controlled by the first readout result. The generated differential voltage signals V_1_ and V_2_ are then delivered to the Self-bias Voltage Comparator. It consists of two inverters and a pair of complementary MOSFET. The inverters composed of PM7 and NM7 are used to invert differential voltage signal V_1_. Additionally, the inverted V_1_ shifts the threshold of the second inverter, which is composed of PM8 and NM8, to the opposite direction by controlling the working state of PM6 and NM6. Then, the second inverter can respond more quickly to the change of differential voltage signal V_2_ and output the final result. The Readout Inverter is used to reverse the output of the Self-bias Voltage Comparator and recover the electrical level of the output signal.

## 4. Experimental Results

In this section, the experiment results of the 4 M 2-bit/cell PCM chip with the assistance of automatic test equipment (ATE) are presented. A brief discussion of the different program pulses for four resistance levels and the comparison with the result of write and verify scheme are then given. Following this, the Resistance Drift, which is the most dominant issue that hinders MLC functionality in PCM, is demonstrated on the basis of the test results. Finally, some simulation diagrams of the program and readout circuits are displayed.

### 4.1. The Resistance Distribution of 2-Bit/Cell Phase Change Memory

**Figure 10:**
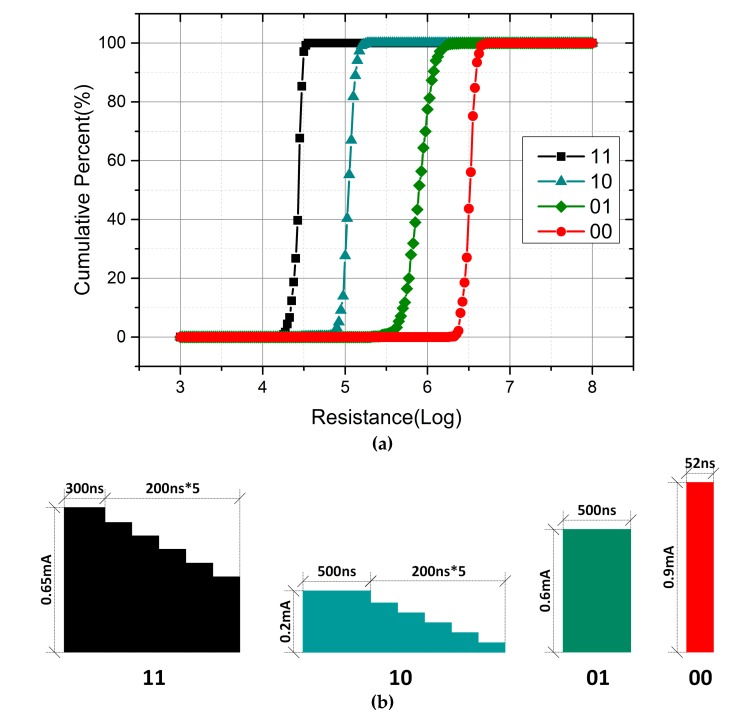
(**a**) Resistance distribution of four states in phase change memory (PCM) cells with a ramp-down current pulse scheme; (**b**) the corresponding program pulses.

Figure 10 shows the resistance distribution of four states in PCM cells and the corresponding program pulses. After RESET initialization, the PCM cells are programmed with different shaped current pulses, including rectangular and ramp-down current pulses, by adjusting the configuration parameters. Almost all the resistance distribution within the range of PCM cell resistance variation can be obtained through this approach. As shown in Figure 10b, the optimal RESET pulse is a rectangular current pulse with an amplitude of 0.9 mA and a width of 52 ns. A current pulse with a larger amplitude cannot increase the resistance of the “00” state, but will result in more power consumption. Additionally, the width of 52 ns is sufficient enough to operate all well-performing cells to “00”. To program the RESET cell to the “01” state, a rectangular current pulse with a smaller amplitude and larger width is performed. For the two states with a lower resistance value, complete crystallization of the PCM cells can be achieved with ramp-down current pulses of different amplitudes. In fact, ramp down pulses with four steps are enough to program the memory cells to their states and the extra two steps are added to achieve a better consistency.

**Figure 11:**
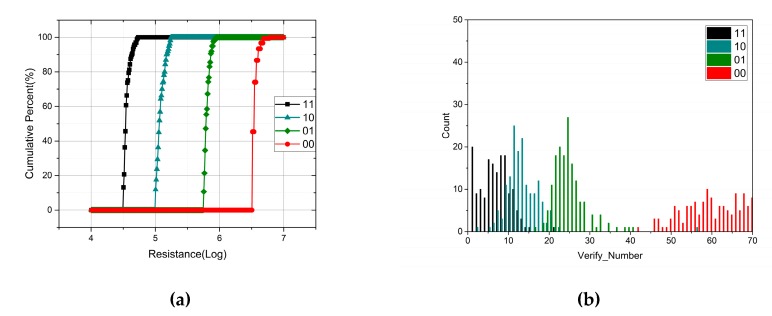
(**a**) Resistance distribution of four states in phase change memory (PCM) cells with the write and verify scheme; (**b**) the number of iterations.

As a contrast, another program scheme based on write and verify is processed with the assistance of ATE. Unlike the previous scheme, this approach starts with an SET operation and then melting rectangular pulses of varying amplitudes in the partial-RESET regime are utilized to increase the resistance. After each program operation, the cell resistance will be readout to verify. If the cell resistance has reached the expected level, the program operation is completed. Otherwise, a rectangular current pulse with a larger amplitude will be used to program until the cell resistance reaches the expected range. In order to compare the two schemes, the resistance range of four states is set as shown in Figure 10a. Figure 11 displays the resistance distribution and the iteration times of four states. To make sure that the cell resistance reaches the expected range accurately, the amplitude increment of the current pulse in each iteration cannot be too large. Consequently, the number of iterations is positively related to the target resistance. For the “00” state with a high resistance, there are over 70 iterations. Note that each iteration includes a read and write operation. Therefore, even though the write and verify scheme improves the consistency of the resistance distribution, the cost of operation time and power consumption is unacceptable. In addition, if the whole scheme is integrated in the chip, the design of the circuit will become more complicated. In conclusion, the scheme of a single-pulse program is preferred in terms of the operation time, power consumption, and cost.

### 4.2. Resistance Drift

**Figure 12:**
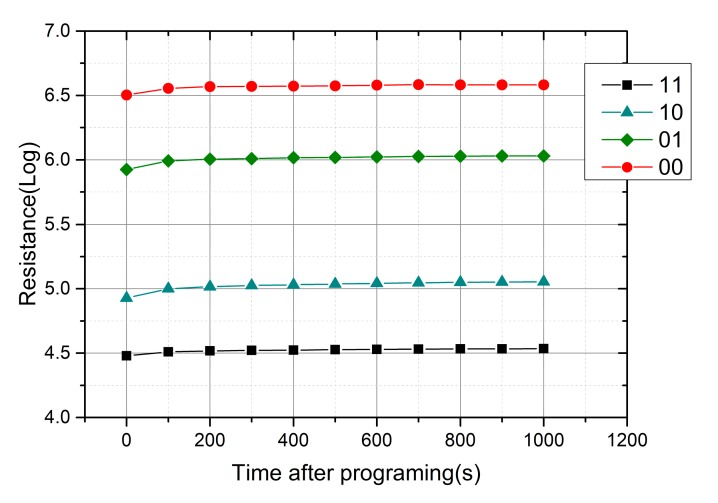
Measurements showing the multilevel drift behavior for a 1000 s time frame.

Amorphous materials are known to display structure relaxation (SR), which is the atomistic-scale rearrangement of an amorphous structure. The amorphous GST in PCM cells also suffers from this phenomenon, resulting in an increase of the electrical resistance with time [13]. As a novel storage technology that improves the capacity at the expense of performance, MLC storage in PCM faces reliability problems as resistance drift reduces the separation between adjacent levels. To study the effect of resistance drift on data retention in memory cells, the resistance variation of PCM cells is recorded within 1000 s after programming. As shown in Figure 12, resistance drift mainly occurs within 100 s after programming. After that, the resistance still increases a little with time, but the separation is enough to distinguish four states.

### 4.3. Simulation Results

**Figure 13:**
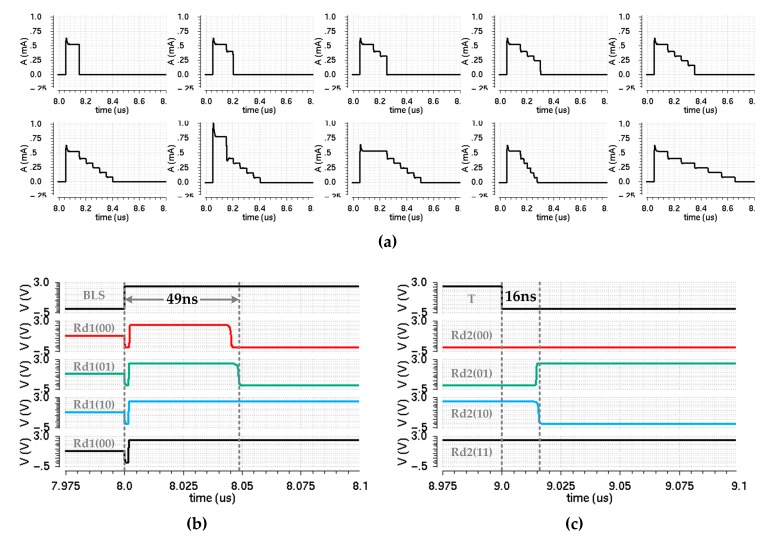
(**a**) Simulation graphs of the ramp-down current pulse generator circuit; (**b**,**c**) readout simulation results of four states in the phase change memory (PCM) cell during the two read operations.

Figure 13a shows the simulation graphs of the ramp-down current pulse generator circuit, and the shape of each pulse corresponds to the design scheme in Figure 7. Figure 13b,c show the readout simulation results of four states in the PCM cell during the two read operations. Taking the worst case into consideration, the final readout time is 65 ns.

## 5. Conclusions

A 2-bit/cell phase change memory chip is presented in this paper with a speed-up write operation. The program scheme adopted in this paper is started with the initialization of memory cells. Then, different shaped pulses, which are produced by the programmable ramp-down current pulse generator, are applied to the addressed cells and program them to the target level. The read operation of the 2-bit/cell is accomplished by a specially designed fully differential read circuit with an optional reference current source. The final results of the simulation and experiment verify the feasibility of the scheme and the functionality of multi-level storage in PCM.

As a comparison, Table 1 summarizes some information and the performance of the chips proposed in this paper and [2]. Our work improves the write and read speed for 2-bit MLC PCM by 6.25 times and 4.9 times, respectively, and decreases the write time from 9.7 μs to <1.6 μs and read time from 320 ns to 65 ns. The omission of the write & verify process reduces not only the number of generated pulses for each bit, but also the power consumption during the programming. Furthermore, the ADCs (analog-to-digital converters) and DACs (digital-to-analog converters) that are necessary for the chip in [2] are dismissed in the new scheme, which greatly cuts down the complexity and cost of the chip design. Therefore, compared with the write and verify scheme, the scheme proposed in this paper is more attractive because of its advantages in speed, power consumption, and cost. 

## Figures and Tables

**Table 1:** Summary of the chips proposed in this paper and [2].

Chips	Chip Proposed in This Paper	Chip Proposed in [2]
CMOS Technology		
Node	SMIC 40 nm	90 nm
Supply Voltage	2.5 V	Digital: 1.2 V Phase change memory (PCM) and analog: 2.5–3.0 V
PCM Cell Array		
Material	C-GST	Doped GST
Access Device	NMOS	NMOS
Cells	4 M cells, 16 accessed in parallel	256 M cells, 16 accessed in parallel
Write		
Access Time	RESET 52 ns+SET 1.5 μs @ 2 bits/cell	9.7 μs @ 2 bits/cell
Program Scheme	Programmable ramp down current pulse	Open-loop single shot, or closed-loop write and verify with one ADC and two DACs integrated in the chip
Readout		
Access Time	65 ns @ 2 bits/cell	320 ns @ 2 bits/cell
Read Scheme	Fully differential read circuit with optional reference current source	1 bit range+6-bit ADC

## References

[1] Kim I.S., Cho S.L., Im D.H., Cho E.H., Kim D.H., Oh G.H., Chung C.H. High performance PRAM cell scalable to sub-20nm technology with below 4F2 cell size, extendable to DRAM applications. Proceedings of the 2010 Symposium on VLSI Technology.

[2] Close G.F., Frey U., Morrish J., Jordan R., Lewis S.C., Maffitt T., Eleftheriou E. (2013). A 256-Mcell Phase-Change Memory Chip Operating at 2+Bit/Cell. IEEE Transactions on Circuits and Systems I: Regular Papers.

[3] Nandakumar S.R., Boybat I., le Gallo M., Sebastian A., Rajendran B., Eleftheriou E. Supervised learning in spiking neural networks with MLC PCM synapses. Proceedings of the 2017 75th Annual Device Research Conference (DRC).

[4] Lee J., Lim D., Jeong H., Ma H., Shi L. (2019). Exploring Cycle-to-Cycle and Device-to-Device Variation Tolerance in MLC Storage-Based Neural Network Training. IEEE Transactions on Electron Devices.

[5] Nirschl T., Philipp J.B., Happ T.D., Burr G.W., Rajendran B., Lee M.H., Joseph E. Write Strategies for 2 and 4-bit Multi-Level Phase-Change Memory. Proceedings of the 2007 IEEE International Electron Devices Meeting.

[6] Close G.F., Frey U., Breitwisch M., Lung H.L., Lam C., Hagleitner C., Eleftheriou E. Device, circuit and system-level analysis of noise in multi-bit phase-change memory. Proceedings of the 2010 International Electron Devices Meeting.

[7] Papandreou N., Pozidis H., Pantazi A., Sebastian A., Breitwisch M., Lam C., Eleftheriou E. Programming algorithms for multilevel phase-change memory. Proceedings of the 2011 IEEE International Symposium of Circuits and Systems (ISCAS).

[8] Papandreou N., Sebastian A., Pantazi A., Breitwisch M., Lam C., Pozidis H., Eleftheriou E. Drift-resilient cell-state metric for multilevel phase-change memory. Proceedings of the 2011 International Electron Devices Meeting.

[9] Stanisavljevic M., Athmanathan A., Papandreou N., Pozidis H., Eleftheriou E. Phase-change memory: Feasibility of reliable multilevel-cell storage and retention at elevated temperatures. Proceedings of the 2015 IEEE International Reliability Physics Symposium.

[10] Raoux S., Burr G.W., Breitwisch M.J., Rettner C.T., Chen Y.C., Shelby R.M., Lam C.H. (2019). Phase-change random access memory: A scalable technology. IBM J. Res. Dev..

[11] Lee K.J., Cho B.H., Cho W.Y., Kang S., Choi B.G., Oh H.R., Park M.H. A 90nm 1.8V 512Mb Diode-Switch PRAM with 266MB/s Read Throughput. Proceedings of the 2007 IEEE International Solid-State Circuits Conference. Digest of Technical Papers.

[12] Bedeschi F., Boffmo C., Bonizzoni E., Resta C., Torelli G., Zella D. Set-sweep programming pulse for phase-change memories. Proceedings of the 2006 IEEE International Symposium on Circuits and Systems.

[13] Ielmini D., Lavizzari S., Sharma D., Lacaita A.L. Physical interpretation, modeling and impact on phase change memory (PCM) reliability of resistance drift due to chalcogenide structural relaxation. Proceedings of the 2007 IEEE International Electron Devices Meeting.

